# Lipid Ligands and Allergenic LTPs: Redefining the Paradigm of the Protein-Centered Vision in Allergy

**DOI:** 10.3389/falgy.2022.864652

**Published:** 2022-05-19

**Authors:** Zulema Gonzalez-Klein, Diego Pazos-Castro, Guadalupe Hernandez-Ramirez, Maria Garrido-Arandia, Araceli Diaz-Perales, Jaime Tome-Amat

**Affiliations:** ^1^Centro de Biotecnología y Genómica de Plantas (UPM-INIA), Universidad Politécnica de Madrid, Madrid, Spain; ^2^Departamento de Biotecnología y Biología Vegetal, Escuela Técnica Superior de Ingeniería Agronómica, Alimentaria y de Biosistemas (ETSIAAB), Universidad Politécnica de Madrid, Madrid, Spain

**Keywords:** allergy, LTP, sensitization, animal models, lipid ligand, allergy

## Abstract

Lipid Transfer Proteins (LTPs) have been described as one of the most prevalent and cross-reactive allergen families in the general population. They are widely distributed among the plant kingdom, as well as in different plant organs ranging from pollen to fruits. Thus, they can initiate allergic reactions with very different outcomes, such as asthma and food allergy. Several mouse models have been developed to unravel the mechanisms that lead LTPs to promote such strong sensitization patterns. Interestingly, the union of certain ligands can strengthen the allergenic capacity of LTPs, suggesting that not only is the protein relevant in the sensitization process, but also the ligands that LTPs carry in their cavity. In fact, different LTPs with pro-allergenic capacity have been shown to transport similar ligands, thus positioning lipids in a central role during the first stages of the allergic response. Here, we offer the latest advances in the use of experimental animals to study the topic, remarking differences among them and providing future researchers a tool to choose the most suitable model to achieve their goals. Also, recent results derived from metabolomic studies in humans are included, highlighting how allergic diseases alter the lipidic metabolism toward a pathogenic state in the individual. Altogether, this review offers a comprehensive body of work that sums up the background evidence supporting the role of lipids as modulators of allergic diseases. Studying the role of lipids during allergic sensitization might broaden our understanding of the molecular events leading to tolerance breakdown in the epithelium, thus helping us to understand how allergy is initiated and established in the individuals.

## Introduction

Prevalence of allergic diseases has been constantly rising along this decade, with clinical reports establishing that up to 3.8% of European and 2.5% of Canadian children are sensitized to at least one food allergen (1, 2). This trend can also be observed in other allergic pathologies, such as asthma, although in this case controversies arise depending on the cohort studied (3). Although allergen specific immunotherapy (AIT) seems a promising tool to manage these diseases in the future, nowadays most allergies are still lacking from a definitive treatment that reverses the sensitized state of the patient (4–6). This results in significant socioeconomical burdens (7), as well as non-allergic comorbidities which range from obesity to mental health disorders (8).

Lipid binding is a characteristic shared by many allergen families, such as Lipid Transfer Proteins (LTPs), Bet v 1-like proteins, 2S albumins, lipocalins, and oleosins among others (9). These bound lipids have been described to have an important role in allergy development; e.g., the lipids of pollen extract induce mast cell chemotaxis, interleukin (IL) 6 release, immunoglobulin E (IgE)-dependent degranulation, and the upregulation of CD1d in dendritic cells (10). Also, the lipids extracted from peanuts can directly activate primary keratinocytes and induce a pro-inflammatory response, showing an increase of *IL6, IL8, TNFA*, and *IL1B* mRNA levels, which is maintained by the allergen (11).

This review focuses on the lipid-binding capacity of LTPs and, in addition, how this binding capacity affects the allergenicity of said protein family. When looking at the bibliography not much information is found, but we will discuss about different studies *in vitro* and *in vivo* that support the idea that the ligands transported by allergenic LTPs are key factors in the sensitization process (12), what might explain why these proteins are associated with severe and potentially lethal anaphylactic reactions (13).

## LTPs as Lipid Binding Proteins

LTPs constitute an important family of food and respiratory allergens. They are defined as small, basic, and thermostable proteins, with a highly conserved structure across the plant kingdom, characterized by a motif of eight residues of cysteine forming four disulfide bonds, and a cavity that allows them to harbor lipids (14). In total, 46 LTPs have been listed as allergens by the World Health Organization (15), being characterized by both their severity and high sensitization rates, especially in the Mediterranean region (16), but also in other countries, such as the UK (17). LTPs are ubiquitously distributed and present cross-reactivity between some members of the protein family, mainly belonging to *Rosaceae* fruits and nuts, thus resulting in elevated numbers of life-threatening reactions due to accidental exposures (18).

Although different classification systems have been proposed, LTPs can be divided in a simple way based on their molecular weight in LTP1s (9-10 kDa) and LTP2s (6-7 kDa) (19, 20). Both have a highly plastic tunnel that can bind a wide range of ligands *in vitro*, such as fatty acids (oleic, linoleic, elaidic or lauric, among others) (21, 22).

## Allergenic LTPs Transport A Unique Kind of Ligand When Studied *IN VIVO*

Nevertheless, when the ligands from several allergenic LTPs purified from natural sources have been studied, it has been proved that all of them share a common characteristic: the native ligand of allergenic LTPs is composed of a camptothecin (CPT)-like polar head bound to a phytosphingosine (PHS) tail: the so-called CPT-PHS ligand. The CPT-PHS ligand was found to be the major molecule complexed to allergenic LTPs *in vivo*. When different LTPs were purified from natural sources, the isolated ligand extracted from their cavities was always the CPT-PHS ligand, except for the case of wheat LTP1 (Tri a 14), for which 2 other different molecules where also found to be bound (12). The mechanism by which the CPT-PHS ligand enters inside the cavity of LTPs needs to be clarified but the actual hypothesis and studies related have been recently reviewed (14). This CPT-PHS ligand has been described to inhibit cell division in the plant and to avoid both pollination and the attack from herbivores (23). Furthermore, the adjuvant activity of the ligand was demonstrated in a mouse model. As it has been previously described for other lipids, the immunogenicity of the CPT-PHS ligand was described to be mediated by CD1d recognition (24, 25).

**Figure 1:**
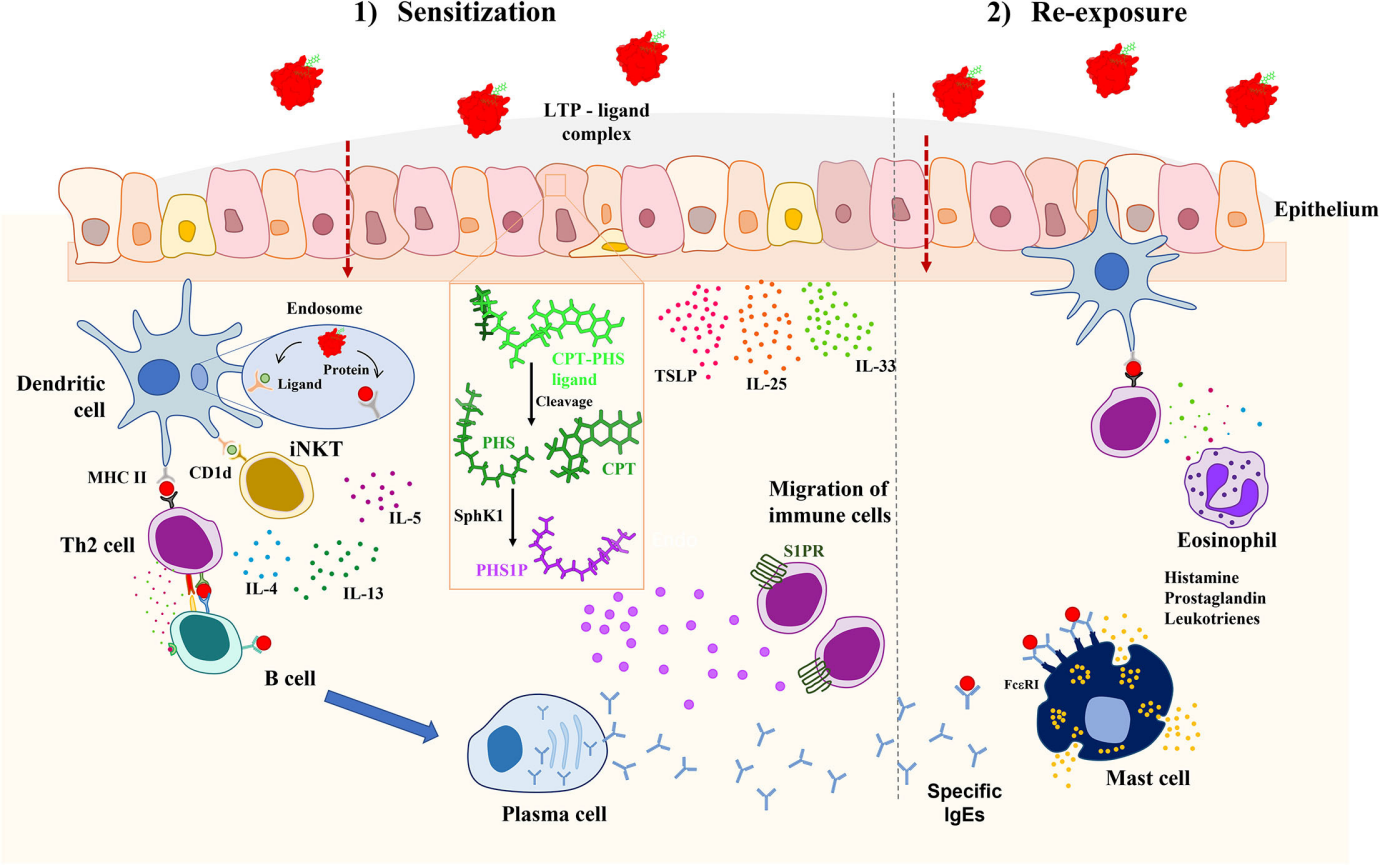
Proposed mechanism of allergic sensitization mediated by LTPs and its associated CPT-PHS ligand *in vivo*. When the complex crosses the epithelium, the CPT-PHS ligand can be both presented by CD1d in dendritic cells to iNKTs, leading to the production of inflammatory cytokines; or converted in phytosphingosine-1-phosphate (PSH1P) inside the epithelial cells, which then promotes the migration of immune cells to the tissue. This explains the adjuvant role of the CPT-PHS ligand, leading to a pro-inflammatory environment in which the protein becomes recognized by the mucosal immune system, promoting the systemic sensitization against it through the production of IgE. CPT, camptothecin; IL, interleukin; iNKT, invariant natural killer T; LTP, lipid transfer protein; MHCII, major histocompatibility complex II; PHS, phytosphingosine; PHS1P, phytosphingosine-1-phosphate; SphK1, sphingosine kinase 1; S1PR, sphingosine-1-phosphate receptor; TSLP, thymic stromal lymphopoietin.

Interestingly, it has been recently discovered that the CPT-PHS ligand can be also metabolized by human cells, converting the PHS tail into phytosphingosine-1-phosphate by human sphingosine kinases. This phytosphingosine-1-phosphate has been demonstrated to mimic some functions of the sphingosine-1-phosphate (S1P) immune mediator, as promoting the migration of immune cells (12). These results are in line with the importance of sphingolipid metabolism in the development of allergic diseases (26) (Figure 1).

## The Importance of Lipids in LTP Sensitization: Conclusions From Mouse Models

Given all the pro-allergenic properties that LTPs can acquire due to lipid binding, as well as the proinflammatory effects exerted by their associated CPT-PHS ligand in human peripheral blood mononuclear cells (PBMCs) (12, 24), it is interesting to assess the conclusions derived from the use of LTPs to induce allergic sensitization *in vivo*, in murine models of the disease. In this context, we will pay special attention to the role played by lipids during the sensitization phase of the allergic response. *In vivo* studies with LTPs are especially relevant, since these proteins are naturally binding lipids (15).

Nevertheless, we have been able to find only two models which take into consideration the lipid binding nature of the allergen (in these cases, peach's Pru p 3), studying the differences in the immune response elicited by the protein alone or in complex with its native ligand, the CPT-PHS ligand (24, 27) (Table 1). Both reports show that Pru p 3 is able to induce anaphylaxis after a skin-based sensitization protocol in mice, both as a protein alone and as a complex with its CPT-PHS ligand. However, when the lipid is present during sensitization, the levels of serological α-Pru p 3 sIgE are significantly higher in C3He/OuJ mice, presumably due to a mechanism involving iNKTs and CD1d-mediated antigen presentation (24). In BALB/c rodents, anaphylaxis was significantly stronger if the lipid ligand was present, although activation of the NLRP3 pathway in the skin was needed prior to allergen addition to induce the allergic phenotype in this strain (27).

**Table 1:** Murine models of sensitization to LTPs.

**Allergen**	**Source**	**Mouse strain**	**Sensitization**	**Adjuvant**	**Challenge**	**Ab response**	**Other observations**	**References**
**Food allergy**							
Api g 2 (LTP1)	Celery	BALB/c	rApi g 2 (10 μg s.c.; once every 2 weeks, for a duration of 8 weeks)	Alum (50 μL Alugel-S)	–	α-Api g 2 sIgE & sIgG (cross-reactive with Art v 3)	–	(28)
Jug r 3 (LTP1)	English walnut	BALB/c	Defatted walnut extract (3 mg i.g.; twice per week, for a duration of 3 weeks)	Cholera toxin (10 μg)	Defatted walnut extract (3 mg i.g.)	α-walnut IgE	–	(29)
Lup an 3 (LTP1)	Blue lupin	C3H/HeJ	Lupin extract (5.7 mg i.g.; days 0, 1, 2, 7, 21 and 28)	Cholera toxin (10 μg)	Lupin extract (5.7 mg i.p.)	α-lupin IgG1 (total IgE was also elevated)	Colonic microbiome composition was heavily modified by lupin allergy	(30)
				No adjuvant		No α-lupin sIgG1 nor total IgE were elevated	Anaphylaxis was not reached under these experimental conditions	(31)
				trypCry1Ab (10 μg)				
Pru p 3 (LTP1)	Peach	C3H/HeJ	rPru p 3 (100 μg e.c.; once per week, for a duration of 6 weeks)	No adjuvant	rPru p 3 (5 μg i.p.)	α-Pru p 3 sIgG1	Strong anaphylactic response post-challenge	(24)
			rPru p 3 (100 μg e.c.) + its natural ligand (10 μg); once per week for a duration of 6 weeks			α-Pru p 3 sIgE & sIgG1		
		BALB/c			rPru p 3 (100 μg i.p.)	No antibody response was observed by ELISA	NLRP3 activation due to skin abrassion prior to allergen addition was needed to achieve the allergic phenotype	(27)
			Pru p 3 (20 μg i.n.; once per week for a duration of 6 weeks)		Pru p 3 (100 μg i.p.)	α-Pru p 3 sIgE	No anaphylactic response post-challenge	(32–34)
				LPS (20 ng)		α-Pru p 3 sIgE & sIgG1	Strong anaphylactic response post-challenge	
**Asthma**							
Art v 3 (LTP1)	Mugwort	BALB/c	rArt v 3 (10 μg s.c.; 6 total immunizations)	Alum (Alugel-S; n.a.)	–	α-Art v 3 sIgG1	–	(35)
Par j 1 (LTP1)	Wall pellitory		rPar j 1 (2 μg i.p.; days 0 and 21)	Alum (2.5 mg Al(OH)_3_)	–	α-Par j 1 sIgE, sIgG1 & sIgG2a	Par j 1 presents an LPS-binding region (Par37) which enhances the antibody response against the allergen	(36)
Par j 1/2 (LTP1s)			rPar j 1 (2 μg) + rPar j 2 (1.65 μg i.p.); days 0 and 21		–	α-Par j 1/2 sIgE, sIgG1 & sIgG2a	–	(37, 38)
Pla a 3 (LTP1)	London plane		rPla a 3 (200 μg i.p.; once per week for a duration of 3 weeks)	Freund's complete adjuvant (n.a.)	Atomized pollen extract (30 min every day for a week)	α-Pla a 3 sIgE & sIgG	–	(39)
Tri a 14 (LTP1)	Wheat		Tri a 14 (10 μg i.p.; days 0, 10, 20 and 30)	Alum (Alhydrogel 3%)	Tri a 14 (10 μg i.n.)	α-Tri a 14 sIgE	T2 cytokine profile and eosinophil infiltration in BALF	(40)

*e.c., epicutaneous; i.g., intragastric; i.n., intranasal; i.p., intraperitoneal; n.a., quantity not available; r, recombinant; s.c., subcutaneous*.

These studies suggest that the immunological properties of LTPs, as in the case of other allergenic proteins such as Bet v 1 (41, 42) or Ber e 1 (43), should not be disengaged from the immunological properties of their physiological ligands, since the contribution of the latter can completely change the landscape of the established response in the organism. Supporting this hypothesis, another report has shown that intranasal sensitization of BALB/c mice to Pru p 3 cannot be achieved with the protein alone, but anaphylaxis is significantly reached when Pru p 3 is co-administered with LPS (32). However, it would be interesting to see if these results could be replicated with the natural ligand of Pru p 3, as well as with other allergenic LTPs frequently reported in food allergy.

In another report, sensitization with whole extracts of blue lupin (major allergen: the LTP Lup an 3) without the use of exogenous adjuvants was not sufficient to induce neither antibody nor anaphylactic responses in C3H/HeJ mice (30, 31), which might contradict the hypothesis that lipid ligands transported by allergenic LTPs have enough adjuvant capacity to induce sensitization against these proteins. However, the route of exposure should be taken into account. In the mentioned article, the lupine allergen alone is delivered via gastrointestinal route, which makes sensitization very difficult. In fact, when attempts have been made to produce food allergy models via the gastrointestinal route in the absence of adjuvants, they have been generally unsuccessful. It is very likely that this pathway is highly polarized toward tolerance. Other models of food allergy use the skin, or even the respiratory tract, as a route of sensitization.

Regarding reports involving mice and LTPs in respiratory allergy, it is also important to address the need to conduct more studies in which sensitization is performed not only against the protein alone, but in conjunction with the lipid it transports, since all the reports that have been published up to now used recombinant allergens to perform the sensitization protocols (Table 1). As a result, the contribution of lipids in the reported responses is dismissed. Still, the studies highlight the relevance that the environment surrounding the protein has in LTP allergy. In the case of Par j 1, *Parietaria judaica*'s major allergen, it is shown that it presents an LPS-binding region that plays an important role in promoting antibody responses against it. As shown by Bonura et al. when BALB/c mice are immunized with a Par j 1 isoform lacking this region, the amount of anti-Par j 1 sIgG1 and sIgG2a produced is significantly lower when compared to whole Par j 1-immunized rodents. Besides, this region seems to be important to antibody binding as well, since mice immunized with whole Par j 1 present significantly lower levels of antibodies against the truncated isoform than to the whole allergen itself (36). Nevertheless, it would be interesting to confirm if these results can be replicated in the presence of *Parietaria*'s Par j 1 native ligand, which has been recently described to have a similar structure to the aforementioned CPT-PHS compound (12).

## From Mice to Humans: The Impact of Lipid Metabolism in LTP Allergy Development

It has become clear that lipids play a fundamental role in the development of allergies, especially to LTPs, but how do they affect the metabolism of allergic patients? Is the lipid metabolism altered in allergic patients from the beginning or are the ligands carried by LTPs able to modify the homeostasis of human lipid metabolism?

Despite the metabolomic information found for other allergies (44–47), there are few studies published in the field regarding specifically LTP allergy. In fact, to date, only two related to the topic can be found. In the first one, the study was performed with food allergic patients in general, but more than 30% of them presented LTP allergy, being one LTP allergic patient the only one who presented severe symptoms. In these food allergic patients, 73 metabolites were significantly altered, including phospholipid-related metabolites. Among them, cortisol, glucose, and some unsaturated lipids were associated with severity (48). On the other hand, the second study directly included the comparison between LTP allergic patients and the control group, but it was based on transcriptome analysis using whole blood cell RNA, and not on metabolomics or lipidomics. Results revealed different expressions in genes related to inflammatory diseases and pathways related to immune regulation. Also, genes related to S1P signaling pathway were exclusively affected in patients with LTP allergy (49).

This is aligned with the important role of sphingolipids in the regulation of mast cells, with S1P as a positive regulator secreted by mast cells after antigen-specific cross-linking of the high-affinity IgE receptor, and sphingomyelin and ceramide acting as negative regulators of mast cell activation (50). Furthermore, S1P is a bioactive signaling molecule with a wide range of functions apart from those related to mast cells, as the regulation of cell proliferation, migration and inflammation (51). These activities and functions are regulated by the S1P receptor signaling system, in which 5 different subtypes of S1P receptors (S1PR1-5) regulate the pathways activated in each case by the S1P, depending also in the tissues or cells implicated, as it has been previously reviewed (52, 53).

These data could also be linked to the recent discovery of the ability of sphingosine kinase human enzyme to metabolize the native CPT-PHS ligand of LTPs and convert the lipid tail of phytosphingosine into phytosphingosine-1-phosphate. Interestingly, phytosphingosine-1-phosphate could mimic the S1P functions and further promote the imbalance of sphingolipid metabolism that seems already altered in allergic patients. This ligand characteristic of LTPs, and the metabolites resulting from its processing, could explain the severity of allergic reactions to LTPs in comparison with other allergenic proteins. However, a more detailed and directed lipidomic analysis of patients allergic to LTPs should be carried out to confirm this hypothesis, since the evidence currently presented is quite indirect. In addition, it would be interesting to make a distinction between the alterations that arose during the first phase of allergic sensitization and the ones occurring during the disease phase, with the aim of discovering new biomarkers that predict in advance the potential development of an allergic response that, especially in the case of LTPs, can have fatal results.

## Conclusions

Although allergy has been classically studied as a protein-centered pathology, few allergens have been described to present intrinsic characteristics that make them more prone to induce type 2 (T2) responses. With allergic diseases increasing in prevalence for the last two decades, there is a growing trend to study allergy as a multifactorial disease (54). In this context, the patients' genetic background and their lifestyle, but also the environmental conditions in which the allergens are found and exposed [such as during an infection (55) or as accompanied by other epithelial stressors (27)] are important variables that must be studied in detail to understand why sensitization ends up happening. LTPs are relevant in this line of study because they are naturally bound to lipidic molecules in the plant (14), thus conditioning that patients are typically co-exposed to both the protein and the lipid when getting in contact with the allergenic source. Hence, LTPs offer a good chance to understand how lipids can modulate the immunological state of the patient, working in conjunction with the allergen itself to induce the atopic phenotype in the organism.

Several mechanisms have been proposed to explain how lipids transported by LTPs can promote allergic sensitization to the protein, including alterations of epithelial barriers integrity and conformational changes that expose IgE epitopes in the protein's surface. However, apart from these indirect effects, it has also been demonstrated that allergenic LTPs share a common CPT-PHS ligand that directly shapes the immunological landscape of the tissue, both in human cell cultures and in *in vivo* mouse models, presumably by altering the sphingolipid metabolism. This is in accordance with recent results obtained by lipidomic techniques to study samples from allergic patients, where clear alterations of phospholipid and sphingolipid homeostasis have been identified.

Nevertheless, despite all the advances accomplished, there is still a great body of work to be done to fully understand how lipids transported by LTPs determine the fate of the immunological response that is going to be initiated toward the protein. Although promising, the number of murine models published up to now regarding this topic is too low. In addition, in the vast majority of them, the use of natural LTP ligands is substituted by exogenous adjuvants, so the contribution of lipid ligands during the sensitization process is dismissed. Similarly, studies derived from lipidomics in profilin allergy and atopic dermatitis suggest that imbalance of lipid metabolic pathways in plasma and skin samples, respectively, are tightly related to allergy pathology and disease severity. However, studies involving LTP allergic subjects are limited, despite the promising results derived from the ones that have already been published in recent years, which highlight the relevance of phospholipids and sphingolipids in the deregulation surrounding the allergic response.

In light of the above, we believe that it is important to design new murine models that can shed some light to the questions that remain unsolved up to this point. Also, we think it could be interesting to encourage LTP patients to participate in lipidomic studies that can help to compare LTP allergy to already well-known similar pathologies, such as profilin sensitization. Globally, results derived from studies about the molecular basis of the LTP allergies could help to understand better the clinical outcomes observed in the patients, as well as to design new therapeutic strategies directed to specific targets to reverse their pathogenic profiles and improve their quality of life.

## Author Contributions

ZG-K and DP-C performed the investigation, wrote the manuscript, and edited the figures and tables. GH-R and MG-A edited the figures. AD-P reviewed the text. JT-A coordinated the investigation and reviewed the text. All authors contributed to the article and approved the submitted version.

## Funding

This work was funded by the Spanish Ministry of Science and Innovation through the project LISENTRA, granted by the Spanish Research State Agency (PID2020-113629RBI00/AEI/10.13039/501100011033). DP-C was granted by Universidad Politécnica de Madrid and Banco Santander for a predoctoral Programa Propio grant. ZG-K and JT-A were funded by the Community of Madrid through the project FOODAL-CM, granted within the co-funded by ESF and ERDF R&D projects call Tecnologías 2018 (S2018/BAA-4574). GH-R was funded by European Commission through the project AllerScreening, granted within the R&D framework programme Horizon2020 (H2020-NMBP-X-KET-2017-768641).

## Conflict of Interest

The authors declare that the research was conducted in the absence of any commercial or financial relationships that could be construed as a potential conflict of interest.

## Publisher's Note

All claims expressed in this article are solely those of the authors and do not necessarily represent those of their affiliated organizations, or those of the publisher, the editors and the reviewers. Any product that may be evaluated in this article, or claim that may be made by its manufacturer, is not guaranteed or endorsed by the publisher.
